# Optimizing locations of waste transfer stations in rural areas

**DOI:** 10.1371/journal.pone.0250962

**Published:** 2021-05-21

**Authors:** Yulong Chen, Zhizhu Lai, Zheng Wang, Dongyang Yang, Leying Wu

**Affiliations:** 1 Key Research Institute of Yellow River Civilization and Sustainable Development & Collaborative Innovation Center on Yellow River Civilization of Henan Province, Henan University, Kaifeng, China; 2 College of Environment and Planning of Henan University, Kaifeng, China; 3 Key Laboratory of Geographical Information Science, Ministry of Education of China, East China Normal University, Shanghai, China; Monash University, AUSTRALIA

## Abstract

Many studies have considered the location of rural waste transfer stations, but most have omitted the impact of transportation network conditions. Traffic accessibility must be considered in optimizing the location of rural waste transfer stations, which is an important difference from the location of rural waste transfer stations. On the basis of previous studies, this study will consider the impact of traffic network on the optimization locations of waste transfer station in the rural areas. The objective of this study was to ensure the minimum Euclidean distance between the waste transfer station and the population center is the maximum, minimize the garbage transportation cost of each population center, construction costs for waste transfer stations, construction and upgrade costs for roads on a traffic network. A multi-objective facility location-network design model and an improved multi-objective simulated annealing algorithm was used to solve the problem. A detailed practical case study was used to illustrate the application of the proposed mathematical model. The results show that transportation network plays an important role in facility location optimization, and the improvement of traffic network conditions can greatly reduce waste transportation costs.

## 1 Introduction

The phrase “Not In My Back Yard” (NIMBY) refers to the well-known social phenomena in which residents oppose the construction or location of undesirable facilities near their home. Examples of such facilities include landfills, waste transfer stations and thermoelectri power plants. NIMBY facilities not only provide convenience or services for residents, but also have a strong negative impact on nearby residents (Saameño Rodríguez, 2005) [1]. The convenience provided by these facilities is shared by the whole society, but the negative impacts may be only borne by some residents closer to them. That’s why NIMBY facilities have been resisted by people. When the distribution of benefits and risks is unfair, there will be dissatisfaction (Xiao, 2010) [2]. Garbage treatment facilities are one of the public facilities with a high degree of evasion.

A good living environment is an indispensable part of realizing the strategy of rural revitalization. In recent years, with the development of rural economy and the continuous improvement of people’s living standards, the amount of domestic waste in rural areas has also increased significantly. The garbage problem in rural areas of China has received far less attention than in urban areas. Garbage treatment in rural areas is also the weak link of the whole environmental governance. In addition, different from urban areas, the location of garbage collection and transfer facilities in rural areas is usually unscientific, and the number of facilities is insufficient. Especially, in rural areas of mountainous environment, low traffic accessibility leads to high cost and low efficiency of waste transportation. This has seriously affected the quality of life of residents in rural areas and brought a serious financial burden to rural development.

In recent years, Chinese government departments began to pay more and more attention to the problem of rural garbage disposal. In November 2015, the Ministry of Housing and Urban-Rural Development and other ten departments jointly issued the "Guiding Opinions on Comprehensively Promoting the Management of Rural Garbage", which proposed that more than 90% of China’s domestic waste will be effectively controlled by 2020. The waste transfer is the key link of garbage disposal. Waste transfer station is the core of the transfer system and plays the role of scheduling link in the system. In addition, garbage collection and transportation costs account for 70% -80% of the entire garbage disposal system (Nie, 2000) [3]. Therefore, how to optimize the location of waste transfer station in rural areas, and plan the garbage collection and transportation route, so as to maximize the efficiency of garbage transfer and reduce the cost of garbage transfer. These are the problems that need to be solved to realize the strategy of Rural Revitalization. Some scholars have studied the location of waste transfer stations.

### 1.1 Relevant NIMBY facilities location models

Several researchers emphasized that the location of waste transfer station should be aimed at minimizing the cost. Anderson (1968) [4] first proposed that the construction of waste transfer stations should follow the concept of “cost optimization”. He stressed that the construction of waste transfer station should ensure the minimum operating cost of the whole waste treatment system. On this basis, Yurteri and Siber (1985) [5] explored the optimal layout of waste transfer stations by constructing a linear programming model, which also laid a theoretical foundation for the location of waste transfer stations. Chang and Lin (1994) [6] optimized the location of waste transfer stations to ensure the minimum total cost of garbage disposal (garbage transportation and facility construction costs, etc.). Chang et al. (1996) [7] studied the optimal locations of waste transfer stations by comprehensively considering economic and environmental benefits. Jia et al. (2006) [8] introduced the concept of reverse logistics system site selection into the location of rural waste transfer stations. First, the set coverage model was used to determine the candidate points of the waste transfer station, and then an integer programming model was built to minimize the operation of the city’s garbage collection and transportation system. Gorsevski et al. (2012) [9] proposed a GIS-based multicriteria analysis for siting a landfill in the Polog region of Macedonia.

Some scholars have explored the optimization of waste transportation routes. Greenberg (1976) [10] conducted an optimization analysis of New York City’s waste transportation routes. Based on Greenberg (1976) [10], Kirca and Erkip (1988) [11] further used an optimized linear model to analyze and discuss the optimal layout of solid waste transfer stations in a certain region of Turkey. Lv et al. (2005) [12] constructed a site selection-path planning problem model and used a two-stage Tabu search heuristic algorithm to solve the problem to obtain the final location and number of waste transfer stations.

Mathematical optimization approaches have been employed for various situations. Du and Guo (2015) [13] constructed a mathematical model for the layout of urban waste transfer stations and proposed a central transfer algorithm for solving the optimal layout of waste transfer stations, and gave the best transfer schemes for garbage in various settlements. Yu and Solvang (2016) [14] proposed an improved mathematical formula to help decision makers choose the location of different facilities, including treatment plants, recycling plants and disposal sites, providing appropriate technologies for hazardous waste treatment. Gu et al. (2017) [15] fully considered the impact of external effects on the location of waste power plants, and established a bi-level planning model to optimize the location of waste power plants in Wuhan. Gallo (2019) [16] proposes a discrete optimization model and a heuristic algorithm to solve the landfill siting problem over large areas. Zhao and Huang (2019) [17] explored the multi-period network design problem is to determine the location of waste facilities in each period during the planning horizon.

### 1.2 Facility location and network design

The classical facility location problems include p-median and p-center problems, the maximum covering location problems and the set covering location problems. In general, classical facility location problems consist of some number of facilities to be located within a given network to satisfy a set of customers in respect of some constrains (Rahmaniani and Ghaderi, 2013) [18].

Travel network design problems address decisions about selecting a number of candidate links between network’s nodes to minimize the sum of construction and travel costs (Cocking, 2008) [19]. Essentially, the facility location-network design problem (FLNDP) is a combination of the facility location and network design problems. Daskin et al. (1993) [20] introduced the first model of FLNDP. Compared with the classical facility location problem, two assumptions are added to the FLNDP. First, the network is not given, and the model determines the configuration of the underlying network. Second, each client is not directly served from facilities and may pass from multiple nodes in the network to get service (Ghaderi and Jabalameli, 2013) [21]. Melkote (1996) [22] identified three types of FLNDPs: the uncapacitated ULNDP (UFLNDP), the capacitated facility location-network design problem (CFLNDP), and the maximum cover facility location-network design problem (MCLNDP). The results of Melkote’s (1996) [22] research were published by Melkote and Daskin (2001a, 2001b) [23, 24].

On the basis of the above research, many scholars have conducted in-depth study on the FLNDP. Cocking (2008) [19] introduced a greedy algorithm, local search, simulated annealing, variable neighborhood search and a branch-and-cut algorithm to solve static budget-constrained facility location network design problems. Bigotte et al. (2010) [25] proposed an optimization model for integrated urban hierarchy and transportation network planning. This model simultaneously determines which urban centers and which network links should be promoted to a new level of hierarchy so as to maximize accessibility to all classes of facilities. Contreras et al. (2012) [26] proposed a combined FLNDP which simultaneously considered the location of facilities and the design of the underlying network so as to minimize the maximum customer-facility travel time; two mixed-integer programming formulations were presented and compared. Contreras and Fernández (2012) [27] also presented a unified framework for solving the general network design problem that combined design decisions to locate facilities and to select links on an underlying network, with operational allocation and routing decisions to satisfy the users demands. Rahmaniani and Ghaderi (2013) [18] presented a mixed-integer model to optimize the location of facilities and the underlying transportation network to minimize the total costs of transportation and operation. The researchers assumed that several types of transfer links existed (each with a different transport capacity, transportation cost and construction cost) and that the links between two nodes could be divided into various types. Ghaderi and Jabalameli (2013) [21] proposed a model for the budget-constrained dynamic (multi-period) uncapacitated FLNDP. They used a greedy heuristic and a fix-and optimize heuristic based on simulated annealing together with “branch and bound” and cutting methods to solve the model. Sadat Asl et al. (2020) [28] dealt with fuzzy capacitated facility location-network design model which aims to select the facilities and candidate links in a way that yield to minimize the total costs, containing: costs of opening facilities, link construction and transportation costs.

Through the literature review, it is not difficult to find that most of the previous studies have focused on the location of waste transfer stations in cities, which can not effectively solve the layout problem of waste transfer stations in rural areas. Different from urban areas, the level of traffic accessibility in rural areas is usually low, and the roads are rugged. This directly leads to higher garbage transportation costs, which adds additional financial burden to local government departments that had limited financial expenditure. Therefore, in rural areas, the reasonable layout of waste transfer stations and the construction or upgrading of roads can be considered to improve the accessibility of roads, so as to achieve the goal of improving the efficiency of waste transportation, reducing transportation costs and improving the quality of life of rural residents. The facility location network design method not only considers the location of facilities, but also considers the optimization of road network. This method can be used to solve the problem of optimal layout of rural NIMBY facilities.

## 2 Model construction

### 2.1 Problem definition and assumptions

Three factors should be considered in the optimization of the location of waste transfer station. On the one hand, a certain distance must be maintained between the waste transfer station and the settlement; the second aspect is to ensure that the total cost of garbage transportation in all settlements is minimum; the third aspect is to ensure that the construction cost of waste transfer station and road upgrade costs are minimal. In this study, the anti-center model in the NIMBY model is used to describe the NIMBY problem of rural waste transfer stations. The anti-center model indicates that there is always a demand point (residential point) closest to the waste transfer station during the location of the waste transfer station. Then, if the distance between the nearest waste transfer station and the residential area (population center) is the longest, it is ensured that all waste transfer stations should be as far away from the demand points as possible. The impact of the waste transfer station on each settlement will be reduced to a minimum. Therefore, the objective function of the anti-center model is selected as one of the objective functions of the multi-objective neighboring facility location design model. Three models were formulated: (1) under the condition that existing roads can’t be upgraded and new roads can’t be constructed, the optimal location of waste transfer stations is determined (Model 1); (2) under the condition that existing roads can be upgraded, the optimal location of waste transfer stations is determined (Model 2); (3) under the condition that existing roads can be upgraded and new roads can be constructed, the optimal location of waste transfer stations is determined (Model 3).

Different from the classical facility location problem, the nodes in the location problem of NIMBY facilities include alternative facility nodes and demand nodes, i.e. demand nodes and the facility nodes can not overlap each other. In addition, demand nodes are divided into pure demand points and transit nodes. The transshipment nodes mean that the node itself must be the demand point, and all inbound demand is transshipped to be served elsewhere. Pure demand nodes only have outflow and no inflow.

It is also necessary to put forward some assumptions: (1) each node of network represents a residence areas or an alternative facility; (2) each node cannot be both a facility and a demand point, that is, a waste transfer station cannot be built on a residential site; (3) waste transfer stations must be located at the alternative facility nodes; (4) At most one waste transfer station can be built on each alternative facility node; (5) the transportation cost between population centers and waste transfer stations is symmetric; (6) There is no capacity limit for the waste transfer station and the road network link; (7) the garbage in each population center is transported as a whole and cannot be split.

### 2.2 Notations

The sets, parameters, and decision variable used in the proposed model are defined (see Table 1).

**Table 1:** The notations of the proposed problem.

**Sets**	
*N*	Set of demand (residential) nodes in the road network, *k*∈*N*
*F*	Set of facility nodes (including new waste transfer stations and existing waste transfer stations)
*M*	Set of alternative waste transfer station nodes in the road network, *m*∈*M*
*O*	Set of all nodes in the road network, *h*,*q*∈*O* = *M*∪*N*
*L*	Set of existing and new candidate transfer links in the network
*L*’	Set of existing road links in the initial network, *L*’∈*L*
**Parameters**	
*d*_*k*_	Demand of population center *k* i.e. the output of garbage
*f*_*m*_	Cost of constructing a waste transfer station at node *m*
*c*_*hq*_	Cost of building or upgrading road links (*h*,*q*)
*tr*_*hq*_	Cost of transporting garbage on the road links (*h*,*q*)
$tr_{hq}^{k}$	The transportation cost of the garbage produced by the settlement *k* on the road links (*h*,*q*), and $tr_{hq}^{k}=tr_{hq}^{}d_{k}$
*D*^*km*^	Euclidean distance from demand point *k* to waste transfer station
*E*_*hq*_	$\left\{ \begin{array}{l} 1\;If\;the\;road\;link\;(h,q)\;exists\;\mathrm{in}\;\mathrm{the}\;\mathrm{intial}\;\mathrm{network}\\ 0\;\mathrm{otherwise} \end{array}\right.$
**Decision Variables**	
*Z*_*m*_	$\left\{ \begin{array}{l} 1\;When\;the\;facility\;\left(waste\;transfer\;station\right)\;is\;arranged\;at\;the\;m\;node\\ 0\;\mathrm{otherwise} \end{array}\right.$
$X_{hq}^{up}$	$\left\{ \begin{array}{l} 1\;If\;the\;road\;link\;(h,q)\;\mathrm{is}\;\mathrm{upgraded}\;\mathrm{and}\;E_{hq}=1\\ 0\;\mathrm{otherwise} \end{array}\right.$
$X_{hq}^{new}$	$\left\{ \begin{array}{l} 1\;If\;the\;road\;link\;(h,q)\;\mathrm{is}\;a\;\mathrm{new}\;\mathrm{link}\;\mathrm{and}\;E_{hq}=0\\ 0\;\mathrm{otherwise} \end{array}\right.$
*X*_*hq*_	$\left\{ \begin{array}{l} 1\;If\;the\;road\;link\;(h,q)\;exists\\ 0\;\mathrm{otherwise} \end{array}\right.$
*E*_*hq*_	$\left\{ \begin{array}{l} 1\;If\;the\;road\;link\;(h,q)\;exists\;(Including\;the\;construction\;of\;upgraded\;roads\;and\;existing\;roads)\\ 0\;\mathrm{otherwise} \end{array}\right.$
$Y_{hq}^{k}$	$\left\{ \begin{array}{l} 1\;The\;garbage\;generated\;in\;population\;center\;k\;needs\;to\;pass\;through\;the\;road\;link\;(h,q)\;\\ 0\;\mathrm{otherwise} \end{array}\right.$
$W_{m}^{k}$	$\left\{ \begin{array}{l} 1\;\mathrm{If}\;the\;garbage\;generated\;in\;the\;population\;center\;k\;\mathrm{is}\;\mathrm{treated}\;\mathrm{by}\;\mathrm{facility}\;m\\ 0\;\mathrm{otherwise} \end{array}\right.$
$Y_{hq}^{h}=E_{hq}$	Shipping out the demand at node *h* using an outbound link is equivalent to constructing that link

### 2.3 Model formulation

Regarding to the above assumptions and notations, the three multi-objective optimization models for the waste transfer stations location network design problem can be defined. Model 1 is described as follows:

(Model 1)

$$Dis\tan ce=Max_{i\in N}\{Min_{m\in M}(D^{km}W_{m}^{k})\}$$ (1)

$$Travel=Min\sum_{k}\sum_{(h,q)\in L^{'}}Rtr_{hq}^{k}Y_{hq}^{k}E_{hq}$$ (2)

$$Cost=Min\sum_{m\in M}f_{m}Z_{m}$$ (3)

subject to

$$\underset{m\in O}{\sum }W_{m}^{k}=1,\;\forall k\in N$$ (4)

$$\underset{k\in N\cdot h\neq k}{\sum }\underset{q\in N}{\sum }E_{qh}Y_{qh}^{k}d_{k}+d_{h}=\underset{k\in N}{\sum }\underset{q\in N}{\sum }E_{hq}Y_{hq}^{k}d_{k},\;\forall h\in N,h\notin F$$ (5)

$$W_{m}^{k}\leq Z_{m},\;\forall k\in N,m\in M:m\neq k$$ (6)

$$Y_{hq}^{k}\leq E_{hq},\;\forall k\in N,(h,q)\in L^{'}$$ (7)

$$Y_{hq}^{h}+Y_{qh}^{q}\leq 1,\;\forall h,q\in N,(h,q)\in L^{'}$$ (8)

$$\underset{m\in M}{\sum }Z_{m}=p$$ (9)

$$Z_{m}\in \left\{0,\;1\right\},\forall m\in M$$ (10)

This model contains three objective functions: formulas (1), (2) and (3). Formula (1) indicates that the minimum Euclidean distance from the waste transfer station to the population center is the largest. Here, the impact of the waste transfer station on the residents of each settlement can be regarded as the point-to-point influence in the space, which has nothing to do with the road. Thus, the Euclidean distance is used to express this effect. The greater the Euclidean distance, the smaller the negative impact of the waste transfer station on the settlement. Formula (2) is to minimize the cost of garbage transportation from each settlement to the waste transfer station. Formula (3) means to minimize the construction cost of waste transfer stations, that is, to minimize the number of newly built waste transfer stations. Formulas (4)–(11) are constraint functions, where formula (4) indicates that the garbage generated in the population center *k* must be transported to a waste transfer station for treatment; formula (5) guarantees the conservation of flow, that is, the inflow of the node is equal to outflow. The node *i* is the transit node. The first item $\sum_{k\in N:h\neq k}\sum_{q\in N}E_{qh}Y_{qh}^{k}d_{k}$ represents the total inflow of garbage produced by the population center *k* to the population center h through the transit population center *q* or directly (*q* = *k*). The second item *d*_*h*_ represents the waste output (demand) of the population center *h* itself. The sum of the first and second terms represents the total inflow of the *h* node. The $\sum_{k\in N}\sum_{q\in N}E_{hq}Y_{hq}^{k}d_{k}$ on the right side of the equation indicates that the garbage produced by the *k* and *h* population centers flows out of the *h* population center to other waste transfer stations for treatment. Eq (6) ensures that the waste can be treated only at the alternative point where there is a waste transfer station; Eq (7) indicates that garbage transportation only exists on the road links that have been created; Eq (8) ensures that there is a one-way link between the nodes, because if the garbage is transported from the population center *h* to the population center *q*, it will no be transported from the population center *q* to the population center; Eq (9) indicates that the number of new waste transfer stations is *p*; In formula (10), *Z*_*m*_ indicates whether there is a new waste transfer station at the alternative facility *m*.

Model 2 is described as follows:

(Model 2)

$$\mathrm{Dis}\tan\mathrm{ce}={\mathrm{Max}}_{i\in N}\left\{{\mathrm{Min}}_{m\in M}\left(D^{kn}W_{m}^{k}\right)\right\}$$ (11)

$$\mathrm{Travel}=\mathrm{Min}\underset{k}{\sum }\underset{(h,q)\in L^{'}}{\sum }Rtr_{hq}^{k}Y_{hq}^{k}X_{hq}$$ (12)

$$\mathrm{Cost}=\mathrm{Min}\underset{(h,q)\in L^{'}}{\sum }Rc_{hq}X_{hq}^{up}+\underset{m\in M}{\sum }f_{m}Z_{m}$$ (13)

subject to

$$\underset{m\in O}{\sum }W_{m}^{k}=1,\;\forall k\in N$$ (14)

$$\underset{q\in O}{\sum }Y_{qh}^{k}X_{qh}=W_{h}^{k}+\underset{q\in O}{\sum }Y_{hq}^{k}X_{hq},\;\forall h\in O,k\in N:h\neq k$$ (15)

$$W_{m}^{k}\leq Z_{m},\;\forall k\in N,m\in M:m\neq k$$ (16)

$$Y_{hq}^{k}\leq X_{hq},\;\forall k\in N,(h,q)\in L^{'}$$ (17)

$$Y_{hq}^{h}+Y_{qh}^{q}\leq 1,\;\forall h,q\in N,(h,q)\in L^{'}$$ (18)

$$\underset{m\in M}{\sum }Z_{m}=p$$ (19)

$$X_{hq}=\max\left\{X_{hq}^{up},E_{hq}\right\},\;\forall (h,q)\in L^{'}$$ (20)

$$Z_{m}\in \left\{0,1\right\},\;\forall m\in M$$ (21)

Compared with Model 1, the objective function formula (13) in Model 2 adds the cost of road upgrading.

(Model 3)

$$\mathrm{Dis}\tan ce={\mathrm{Max}}_{i\in N}\left\{{\mathrm{Min}}_{m\in M}\left(D^{km}W_{m}^{k}\right)\right\}$$ (22)

$$\mathrm{Travel}=\mathrm{Min}\underset{k}{\sum }\underset{(h,q)\in L^{'}}{\sum }Rtr_{hq}^{k}Y_{hq}^{k}X_{hq}$$ (23)

$$\mathrm{Cost}=\mathrm{Min}\underset{(h,q)\in L^{'}}{\sum }Rc_{hq}\left(X_{hq}^{up}+X_{hq}^{new}\right)+\underset{m\in M}{\sum }f_{m}Z_{m}$$ (24)

subject to

$$\underset{m\in O}{\sum }W_{m}^{k}=1,\;\forall k\in N$$ (25)

$$\underset{q\in O}{\sum }Y_{qh}^{k}X_{qh}=W_{h}^{k}+\underset{q\in O}{\sum }Y_{hq}^{k}X_{hq},\;\forall h\in O,k\in N:h\neq k$$ (26)

$$W_{m}^{k}\leq Z_{m},\;\forall k\in N,m\in M:m\neq k$$ (27)

$$Y_{hq}^{k}\leq X_{hq},\;\forall k\in N,(h,q)\in L^{'}$$ (28)

$$Y_{hq}^{h}+Y_{qh}^{q}\leq 1,\;\forall h,q\in N,\;(h,q)\in L^{'}$$ (29)

$$\underset{m\in M}{\sum }Z_{m}=p$$ (30)

$$X_{hq}=\max\left\{E_{hq},X_{hq}^{up},X_{hq}^{\mathrm{new}}\right\},\;\forall (h,q)\in L^{'}$$ (31)

$$Z_{m},X_{hq}^{up},X_{hq}^{new}\in \left\{0,1\right\},\;\forall m\in M,h,q\in N$$ (32)

Compared with Model 2, the formula (24) in Model 3 adds the road construction cost. *L* indicates the set of existing and new candidate transfer links in the network. In the constraint conditions, the formula (31) enforces the binary restriction on the link decision variable. It means that if the transfer link from node *h* to *q* may be initial state or upgraded or new constructed.

## 3 Algorithm solution

### 3.1 Multi-objective optimization problem

There are many methods to solve multi-objective optimization problems. These methods can be divided into three types: priori methods, posteriori method, and the interactive methods. The pioneering study on optimality in multi-objective problem is attributed to Pareto (Coello, 1999) [29]. Therefore, multi-objective optimization is usually called Pareto optimization. The main difference between multi-objective optimization and single-objective optimization is that the solution of multi-objective optimization is not unique, but an optimal solution set due to conflict of objective between them. A multi-objective minimization problem can be expressed as following:

$$\begin{array}{c} \min imize\;F(x)=[f_{1}(x),f_{2}(x),\ldots ,f_{n}(x)]\\ s.t.\;x\in \Omega \end{array}$$ (33)

Where Ω indicates the feasible solution area, *f*_1_(*x*),*f*_2_(*x*),…,*f*_*n*_(*x*) represent that *n* objective functions having conflict with each other. Understanding the concepts of Pareto is necessary for solving multi-objective problems.

According to Eq (33), if the feasible solution x* dominates another feasible solution *x*(shown by x*≻*x*), the following two conditions must be satisfied:

$$\begin{array}{l} \forall i\in \{1,2,\ldots ,n\},f_{i}(x^{*})\leq f_{i}(x)\\ \exists k\in \{1,2,\ldots ,n\},f_{k}(x^{*})<f_{k}(x) \end{array}$$ (34)

Where x*,*x*∈Ω.

If there is no another *x*∈Ω satisfies with *x*≻x*, the solution x*∈Ω is Pareto optimal solution. The set of all Pareto optimal solutions is Pareto optimal set (PS).

Pareto optimal front (PF) is the set consisting of objective function vectors related to PS.

$$PF=\{y={\left[f_{1}(x),f_{2}(x),\ldots ,f_{n}(x)\right]}^{T}|x\in PS\}$$ (35)

Therefore, the main task of multi-objective optimization is to find the Pareto optimal solution and get the Pareto front.

### 3.2 The IMOSA algorithm

Simulated annealing (SA) is a local search-based heuristic that can avoid the local optimum solution by accepting inferior solutions during the solution iterations. The SA algorithm is based on general concepts regarding the gradual cooling process of metal. The SA algorithm starts with an initial solution at a high temperature. The temperature is reduced according to a certain temperature regulation process. At each step, a neighbor is generated randomly and its corresponding objective function is calculated. The movement to improve the objective function is always accepted, and other movements are accepted with some probability. The SA algorithm is effective for solving highly complex combinatorial problems. However, compared with the population evolution of a genetic algorithm, the search efficiency of the SA is limited. Therefore, based on the SA algorithm, this study adopted the concepts of group search, mutation and crossover of a genetic algorithm, and combined them with a fast non-dominated sorting approach to develop an improved multi-objective simulated annealing (IMOSA) algorithm. The IMOSA algorithm was then used to solve the multi-objective facility location-network design model.

#### 3.2.1 The framework of IMOSA algorithm

**Fig 1:**
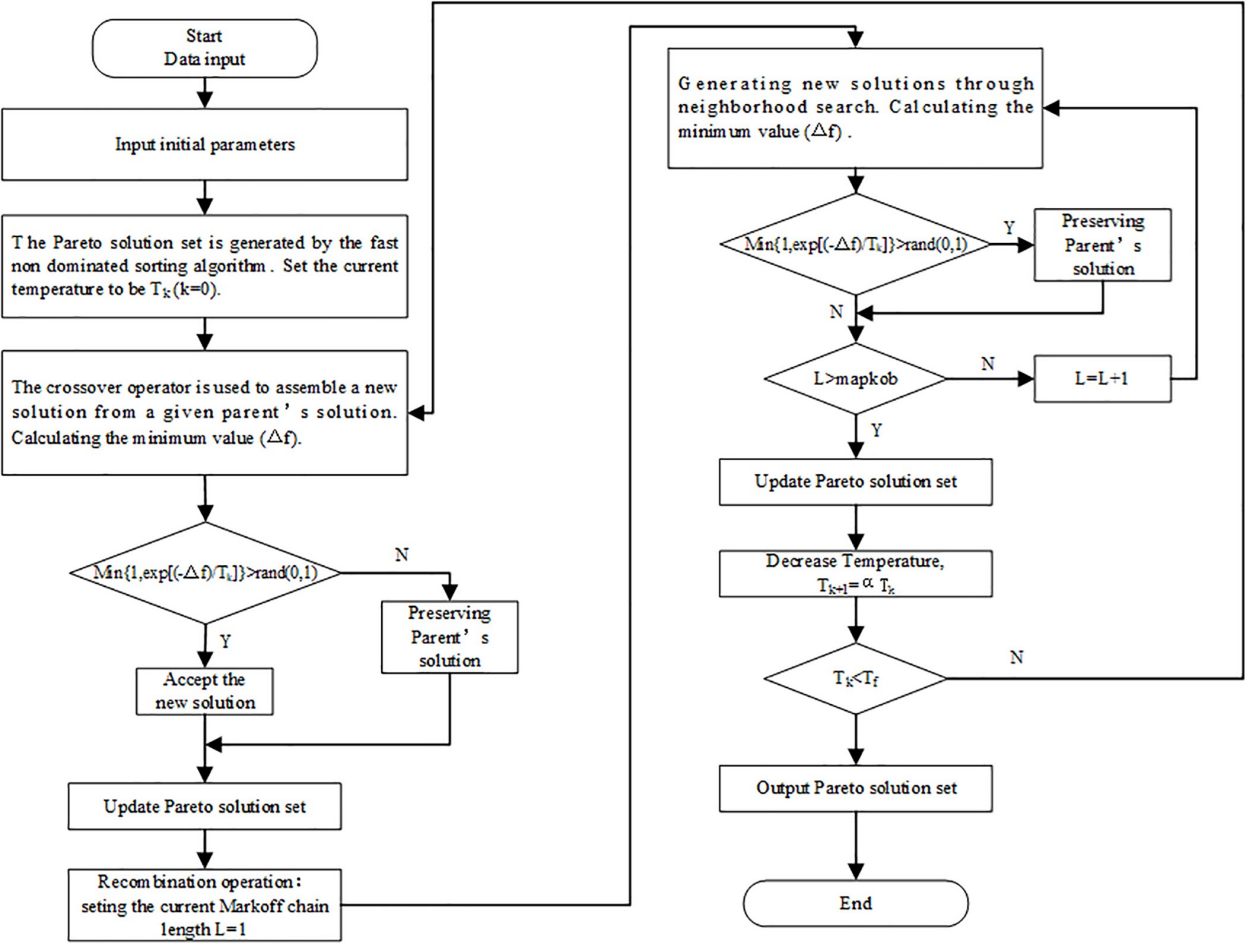
The overall flowchart of IMOSA algorithm.

The main operations of the IMOSA algorithm are shown in Fig 1. In the flow chart, *popsize* represents population size; *T*_0_ denotes the initial temperature; *T*_*k*_ and *T*_*f*_ indicate the current temperature and the end temperature; *α* indicates the cooling coefficient and is used to control the cooling rate; *rand* represents a random number; *mapkob* indicates the length of the Markov chain; *L* represents the current length of the Markov chain; and Δ*f* denotes the minimum value of the difference between the objective function of the new solutions and the parent solution.

The algorithm is started by initializing the population, with each individual in the population representing a solution (location of new waste transfer station, road upgrade and construction scheme). This establishes the values of *X*_*hq*_ and *Z*_*m*_, then the constraints (9)-(10), (19)-(21) and (30)-(32) are naturally satisfied. We use the Floyd algorithm [30] to calculate the shortest distance between any two nodes and record the intermediate nodes passed by. Subsequently, each demand point is assigned to the closest facility. According to this approach, constraints (4)-(8), (14)-(18), (25)-(29) are naturally satisfied and the values of the objective function 1, 2 and 3 can be calculated. The Pareto solution set (distance from settlement to nearest facility based on Euclidean distance, transportation cost and construction cost in each settlement) is generated using the fast non-dominated sorting approach. In the process of gradual cooling, all individuals in the current population are randomly matched as a parent solution. The parent solution generates new solutions via the recombination operations and neighborhood generating operations. The Pareto solution set is continually updated according to the Metropolis rule, and the process is repeated until the current temperature reaches its lowest value and output the final Pareto solution set.

#### 3.2.2 Solution representation

**Fig 2:**
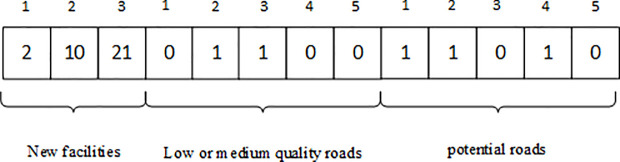
A solution representation.

The solution obtained by the improved multi-objective simulated annealing algorithm is represented by an array. The array elements of this array consist of the location number of new facilities and 0 or 1 number. 1 indicates upgrading of low-quality and medium-quality roads or constructing potential roads. 0 indicates no upgrade or no construct road. Location number of new waste transfer stations, roads to upgrade (low-quality roads and medium-quality roads) and potential roads are arranged in the front, middle and back of the array, respectively. For example, the road network consists of three new waste transfer stations, five roads to upgrade and five potential roads. The solution can be expressed as an array containing 13 elements (as shown in Fig 2). The first three elements of this array indicate that the three new waste transfer stations is located at the node numbered 2, 10 and 21. The fourth to thirteenth elements of the array represent upgrading or constructing second and third low or medium-quality roads, first, second and fourth potential roads, respectively.

#### 3.2.3 Crossover operation and neighborhood generation

The parent’s solutions generate new solutions after crossover operations and neighborhood generating operations. The purpose of crossover operation is to assemble a new offspring from a given parent’s solution. In this algorithm, single point crossover is used to generate new solutions. In addition, by randomly changing any array elements of the parent solution, the offspring solution is obtained. This process is called neighborhood generation. The neighborhood generation is the process of generating new solution and it is one of the main factors that affect the performance of IMOSA algorithm.

#### 3.2.4 Acceptance strategy

After generating a new solution, it should be decided whether the solution is accepted in the next iteration. By calculating the minimum value of the difference between the objective function of the new solutions and the parent’s solution, we can decide whether the new solution is accepted.

#### 3.2.5 Fast non-dominated ranking

We use fast non-dominated sorting method to sort and stratify the solution group of IMOSA algorithm. For the convenience of description, *Pop* is the set of all solution individuals in the group; *n*_*p*_ is the number of other individuals dominating individual *p*, *S*_*p*_ is the set of other individuals dominating individual *p*, and *F*_*i*_ is the *i*th level Pareto front. The calculation process of non-dominated sorting algorithm is as follows: (1) Set first Pareto front to an empty set, *F*_1_ = ∅. (2) For each individual *p* in group *Pop*, set *n*_*p*_ = 0, *S*_*p*_ = ∅. If *p* dominates *q* in group *Pop*, then *q* is added to *S*_*p*_, *S*_*p*_ = *S*_*p*_∪{*q*}; if *q* dominates *p* in group *Pop*, then *n*_*p*_ = *n*_*p*_+1; if there is no other individual in the group that can dominate *p*, then solution individual *p* is added to the first level Pareto front, *F*_1_ = *F*_1_∪{*p*}. (3) Set *i* = 1. (4) Set *F*_*i*_≠∅, *Q* = ∅, *n*_*q*_ = *n*_*q*_−1, *p*∈*F*_*i*_, *q*∈*S*_*p*_, if *n*_*q*_ = 0, then add *q* to *Q*, *Q* = *Q*∪{*q*}. (4) Set *i* = *i*+1, *F*_*i*_ = *Q*, then go to step (4).

### 3.3 Computational experiments

To verify the feasibility and effectiveness of the improved multi-objective simulated annealing algorithm (IMOSA), the proposed algorithm was compared with the classical multi-objective simulated annealing algorithm (MOSA) and multi-objective genetic algorithm (MOGA) through computational experiments. Considering that the Pareto solution set in multi-objective optimization problem is unknown, we use three applied evaluation indicators: Non-dominance Number (NN), Modified Generation Distance (MGD) and Spacing (SP) to evaluate the optimization results [31, 32].

The test problems were generated randomly with the following process: (1) The locations of the 100 network nodes (candidate facilities and demand points) are generated randomly and uniformly distributed over a 100*100 area. (2) There are links between all network nodes. (3) The link travel cost is proportional to the length of the link. (4) The demand of demand nodes and the construction cost of candidate facilities are randomly generated by normal distribution, and the expectation and standard deviation of normal distribution are 100 and 50, respectively.

To eliminate the influence of experimental randomness on the performance evaluation of the algorithm, the IMOSA, MOSA and MOGA algorithm were used to calculate 10 times, respectively, and the final Pareto optimal frontier solution set of the example was obtained. Then, the performance of the three algorithms is compared and analyzed by using the three indicators: NN, MGD, SP. The average and standard deviation of each indicator are obtained by running each algorithm 30 times, and the final calculation results are shown in Table 2.

**Table 2:** Comparisons of performance indicators of two algorithms.

Algorithms	NN		MGD		SP	
	average	standard deviation	average	standard deviation	average	standard deviation
MOSA	14	2.96	1074.74	452.56	3656.54	2914.95
MOSA	25	5.82	635.70	382.71	3165.77	1243.00
IMOSA	42	5.20	507.67	272.69	2155.30	1108.07

Table 2 shows that the IMOSA algorithm has more Pareto solutions than MOSA and MOGA algorithms. Comparing the average and standard deviation of MGD and SP indices obtained by the three algorithms after 30 runs respectively, it shows that IMOSA algorithm is superior to MOSA and MOGA algorithm. In addition, the results of IMOSA algorithm approximate the real situation better, have better convergence and distribution, and the spatial distribution of the target is more uniform.

## 4 Example application

### 4.1 The basic data

**Fig 3:**
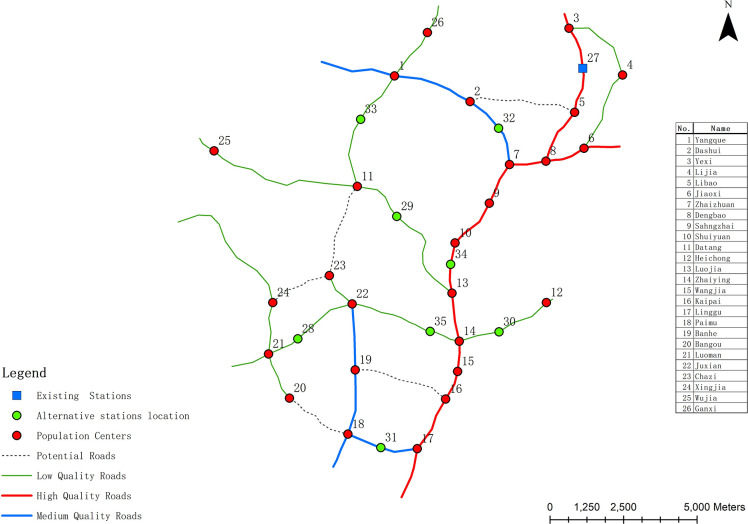
Road network with potential roads in the layout of the waste transfer station.

To test the feasibility and effectiveness of the models and algorithms proposed in this study, a town in Guizhou Province is selected as the research area. Guizhou is located in a mountainous environment that is typical of areas designated for “Rural Revitalization”. There are some problems in the research area, such as the lack of financial funds, the serious lack of waste disposal facilities and so on. In addition, the high cost of garbage transportation brings a great burden to the local financial expenditure. Therefore, it is urgent to select a scientific site for the waste transfer station in the research area. The basic data of the research area used in this research mainly includes road network layers, population centers (natural villages), waste transfer stations and Administrative boundary layer in the research area. As shown in Fig 3, the town consists of 26 villages (nodes, numbered 1 to 26) with a total population of 38,650, and there is already a waste transfer station in the research area. In addition, the population of each population center (natural village) is obtained through field investigation. The 6 potential roads are designed according to the actual topography of the study area in Google Earth. These roads can be divided into three categories according to their quality: high, medium and low. Also, it should be noted that the costs of upgrading or constructing vary according to different types of roads. As a result, the cost of upgrading low and medium-quality roads to high-quality roads is lower than the cost of constructing new roads. In addition, the waste transportation cost per unit weight varies with different qualities of roads. Generally, the waste transfer stations are beside the roads. It is agreed that no waste transfer stations within 500m of the population center. (Chen, 2018). Under the above conditions, 7 alternative facilities were randomly selected on the road network.

### 4.2 Parameter setting

Total daily garbage production by population center is regarded as the demand of each demand point. Assuming that 1.5 kg of garbage is generated per capita per day (Li, 2008) [17], then this value is multiplied by the population of each population center to get the total amount of daily garbage production. In addition, it is assumed that the transportation cost of per kilometer of 1kg waste on high-quality roads is 10, the transportation cost of medium-quality road is 2 times that of high-quality roads, and the transportation cost of low-quality road is 3 times that of high-quality roads. The cost of road construction and upgrading is positively proportional to the road distance. Therefore, the construction cost of a new road, the cost of upgrading low and medium-quality roads to high-quality roads are calculated as 160 times, 80 times and 48 times of road distance (Unit: m) between two population centers, respectively. It is also assumed that the construction cost of each candidate waste transfer station is the same at 600,000. In addition, according to the calculation scale of the model and referring to the existing studies, the initial temperature *T*_0_ is set to empirical value 500; the termination temperature *T*_*f*_ is set to 0.1; the cooling coefficient *α* is set to 0.95; and the Markov chain length *L* is set to 30 at the current temperature.

### 4.3 Calculation results and analysis

#### 4.3.1 Calculation results

The optimal solution of models is obtained by programming in MATLAB with the predetermined value of different parameters. When the number of new waste transfer stations is set to 1, 2, and 3, respectively, the Pareto optimal solution set of Model 1 is obtained. Each solution in the Table 3 represents an optimum location of the waste transfer station. Decision makers can choose from the Pareto optimal solution to select the final scheme according to his own preference. Due to space limitations, only the situation of two new waste transfer stations is described here.

**Table 3:** The corresponding scheme and objective function value of Pareto optimal solution of Model 1.

Solution	Number of new facilities	Location of new facilities	Obj1	Obj2	Obj3
#1	*p* = 1	30	1372	6615	600000
#2	*p* = 1	31	1211	5175	600000
#3	*p* = 2	30,33	1372	6028	1200000
#4	*p* = 2	30,32	1290	5833	1200000
#5	*p* = 2	31,32	1211	4341	1200000
#6	*p* = 2	28,31	1122	4153	1200000
#7	*p* = 2	28,34	739	3772	1200000
#8	*p* = 3	29,30,33	1372	5995	1800000
#9	*p* = 3	30,32,33	1290	5476	1800000
#10	*p* = 3	31,32,33	1211	3984	1800000
#11	*p* = 3	28,31,32	1122	3320	1800000
#12	*p* = 3	28,33,34	739	3304	1800000

**Fig 4:**
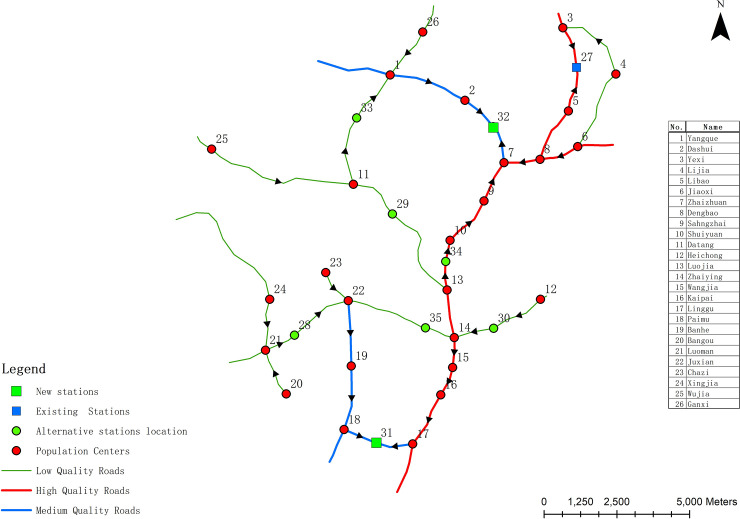
The optimal solutions in Model 1.

Table 3 shows that the corresponding decision-making schemes for the two waste transfer stations in Model 1 are #3 to #7. Since the cost of facilities construction is the same in the five schemes, only the first objective value (maximum minimum NIMBY distance) and the second objective value (garbage transportation cost) are needed to be compared and analyzed in the final decision-making scheme selection. It is not difficult to find that the negative impact on residential area decreases with the increase of waste transportation cost. Therefore, it is necessary for decision makers to choose one of the four Pareto optimal solutions as the final tradeoff solution. According to Table 3, Scheme #3 can ensure low negative impact on residential area, while waste transportation cost is high. Scheme #7 can ensure the low waste transportation cost, but the negative impact on residential area is high. According to the limited financial income of the study area, the waste transportation cost should not be too high while ensuring that the negative impact on residential area is as low as possible. Thus, schemes #4, #5 and #6 may be regarded as tradeoff scheme. As an illustration, scheme #5 was chosen as the final decision-making plan for Model 1. In scheme #5, the candidate locations for the new waste transfer station are No. 31 and No. 32 (*Z* = 31;*Z* = 32). The maximum minimum NIMBY distance is 1211, the garbage transportation cost is 4341 and the construction cost of the waste transfer station facility is 1.2 million. The locations of the new waste transfer stations are shown in Fig 4. As Fig 4 demonstrates, the moving direction of garbage to waste transfer station is shown on roads. Table 4 shows the NIMBY distance and waste transportation cost of each population centers under the facility optimization results of Model 1.

**Table 4:** NIMBY distance and waste transportation cost of population centers in scheme #5 under Model 1.

Population center No.	NIMBY distance(m)	transportation cost	Population center No.	NIMBY distance(m)	transportation cost
1	3965	158	14	4496	46
2	1332	41	15	3678	84
3	1443	48	16	2754	83
4	1372	105	17	1242	20
5	1520	26	18	1211	30
6	2720	189	19	2785	60
7	1290	54	20	3532	446
8	1959	10	21	4973	377
9	2569	158	22	4987	83
10	4179	122	23	6109	178
11	5202	226	24	6152	782
12	6151	298	25	9713	323
13	5787	177	26	4063	218

**Fig 5:**
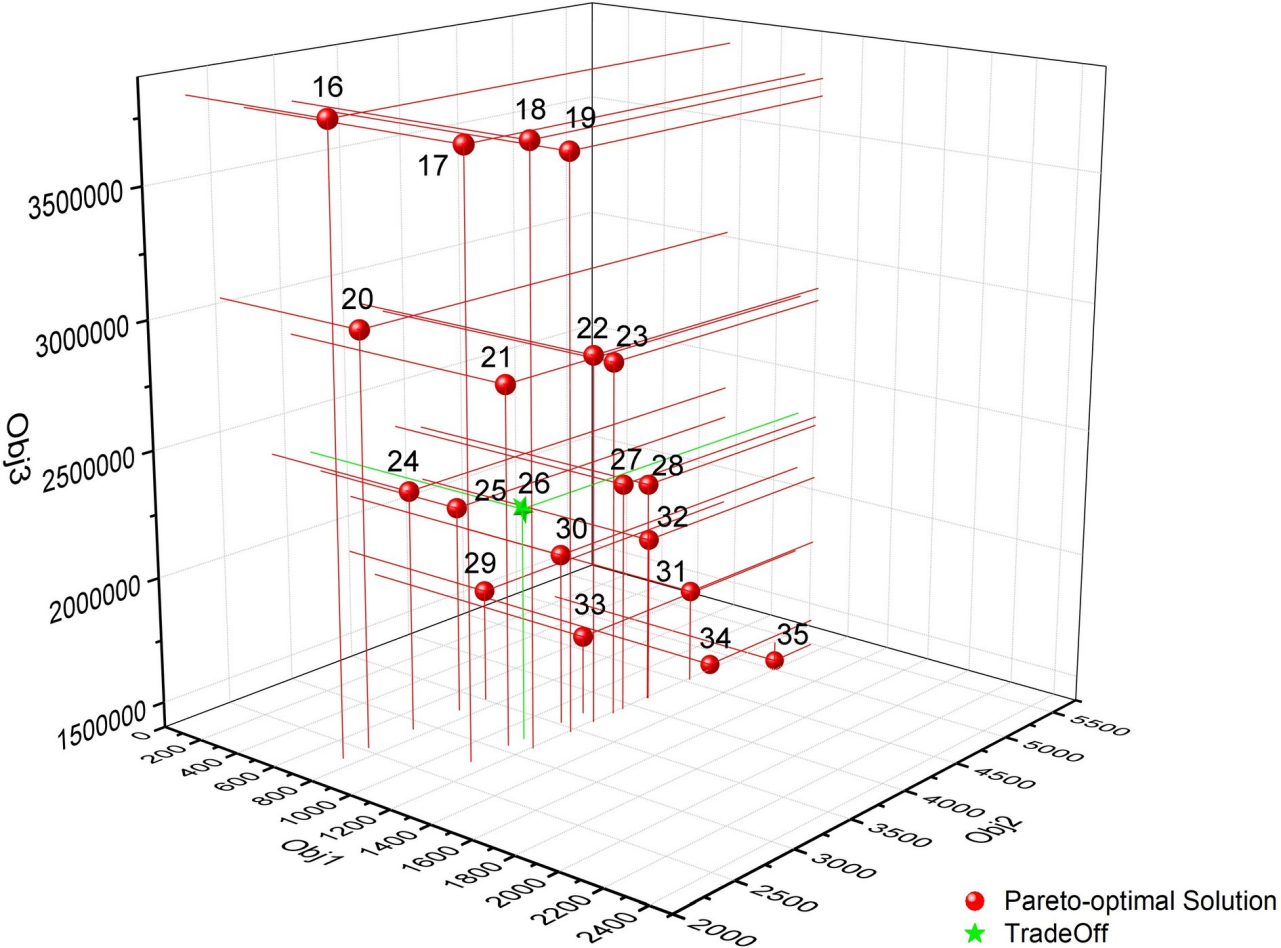
The distribution of the Pareto optimal solution of Model 2 in the three-dimensional coordinate system (p = 2).

**Fig 6:**
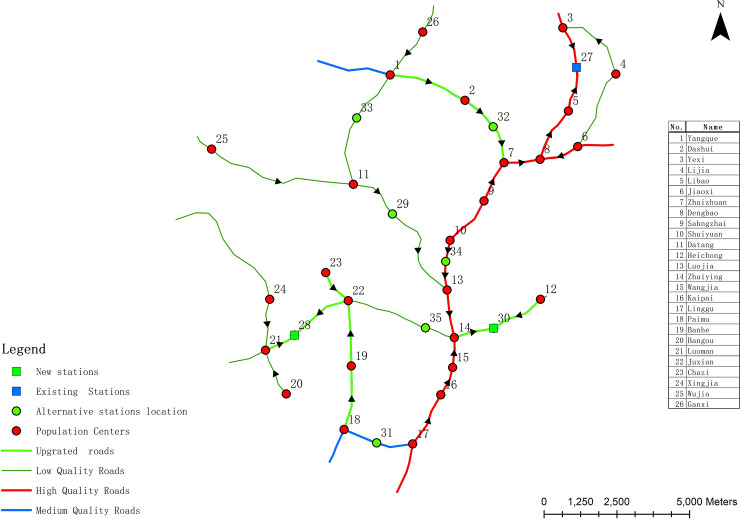
Waste transfer station layout and road upgrading results of scheme #26 under Model 2.

S1 Table shows the Pareto optimal solution set of Model 2 when the number of new waste transfer stations is set to 1, 2 and 3, respectively. Each solution in the table represents an optimal layout scheme. Similarly, after finding the Pareto optimal solution set, the decision-maker can choose the final decision-making scheme according to his own preference. It can be seen from S1 Table that the scheme numbers of the newly built two waste transfer stations are No.16 to No.35. To make it easy for decision makers to judge, the Pareto optimal solutions of the 2 new waste transfer stations is displayed through the three-dimensional coordinate system diagram, as shown in Fig 5. Schemes #34 and #35 can ensure low construction cost, but the total cost of garbage transportation in each population center is high. On the contrary, schemes #16 and #17 can ensure that the total cost of garbage transportation in each population center is low, but the construction cost is high. According to the actual situation of limited local financial revenue in the study area, the decision maker needs to choose one of the 15 Pareto optimal solutions as the final tradeoff solution. Fig 5 shows that schemes #21, #25 and #26 may be regarded as tradeoff scheme. As an illustration, scheme #26 was chosen as the final decision-making plan for Model 2. In Scheme #26, the locations of the new waste transfer stations are located at No.28 and No.30, and 7 roads have been upgraded. The road upgrading and the location of the new waste transfer station in Model 2 are shown in Fig 6. As Fig 6 demonstrates, the moving direction of garbage to waste transfer station is shown on roads. Table 5 shows the NIMBY distance, garbage transportation cost and construction cost of each population center under the optimization results of Model 2.

**Table 5:** The NIMBY distance and waste transportation cost of population centers in scheme #26 under Model 2.

Population center No.	NIMBY distance(m)	transportation cost	Population center No.	NIMBY distance(m)	transportation cost
1	6412	196	14	1392	12
2	4002	113	15	1953	48
3	1443	48	16	2922	89
4	1372	105	17	4855	86
5	1520	26	18	3663	166
6	2720	233	19	2218	79
7	4118	194	20	2038	132
8	3399	13	21	1122	27
9	4393	352	22	2199	33
10	3375	126	23	2407	66
11	6912	205	24	1499	173
12	1891	38	25	7004	306
13	2073	85	26	5430	250

**Fig 7:**
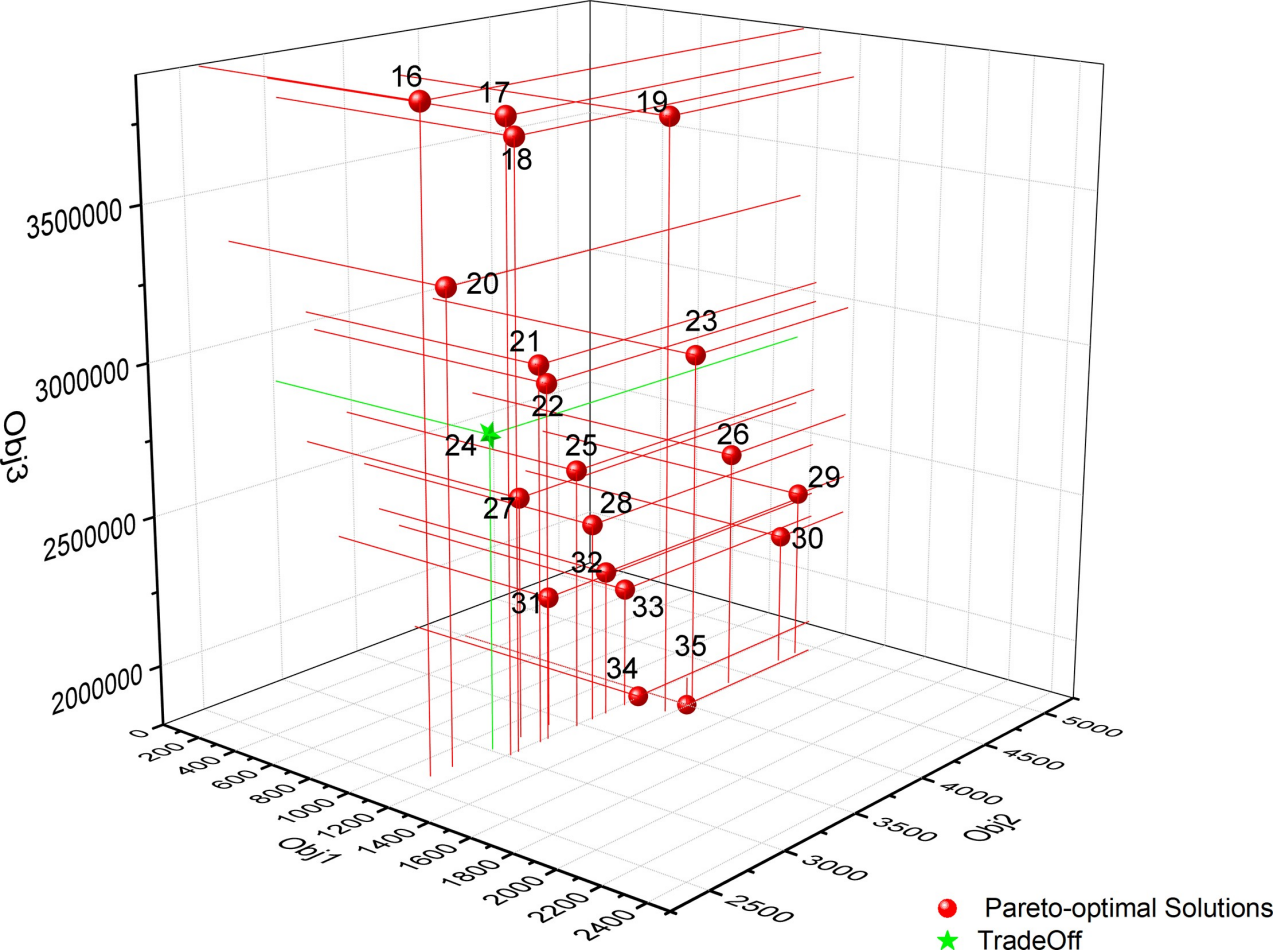
The distribution of the Pareto optimal solution of Model 3 in the three-dimensional coordinate system (p = 2).

**Fig 8:**
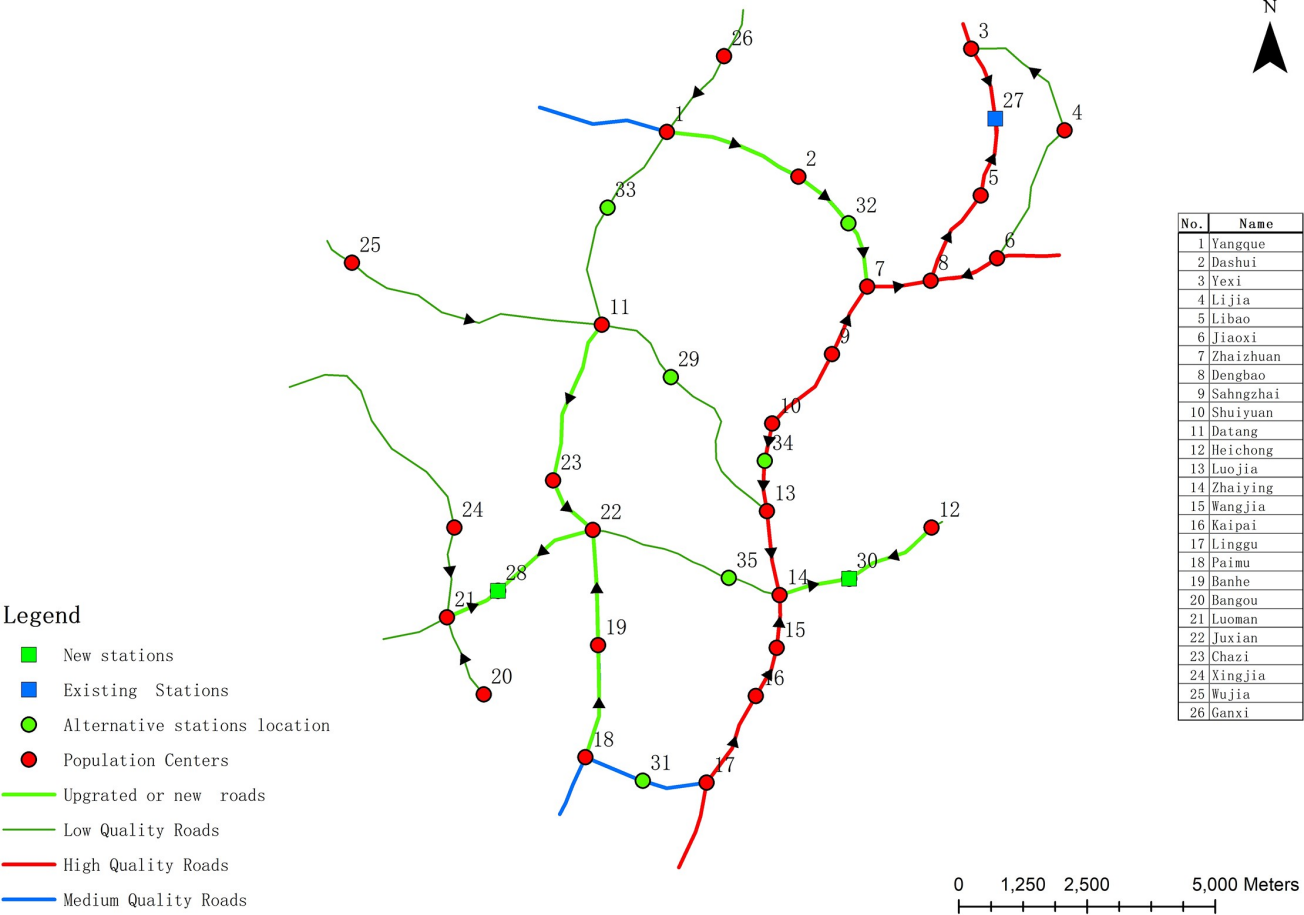
Waste transfer station layout and road upgrading results of scheme #24 under Model 3.

S2 Table shows the Pareto optimal solution set of Model 3 when the number of new waste transfer stations is set to No. 1, No. 2 and No. 3, respectively. As in Model 1 and Model 2, after finding the Pareto optimal solution set, the decision-maker can choose the final decision-making scheme according to his own preference. It can be seen from S2 Table that the scheme numbers of the newly built two waste transfer stations are 16 to 35. As Model 2, to make the decision-maker easy to judge, the Pareto optimal solution of the 2 new waste transfer stations is displayed through the three-dimensional coordinate system diagram, as shown in Fig 7. Schemes #34 and #35 can ensure that the construction cost is small, but the total cost of garbage transportation in each population center is high; schemes #16 and #17 can ensure that the total cost of garbage transportation in each population center is low, but the construction cost is high. According to the actual situation, the decision maker needs to choose one of the 15 Pareto optimal solutions as the final tradeoff solution. It can be seen from Fig 7 that schemes #22, #24 and #25 may all be selected as tradeoff decision schemes. Scheme #24 is chosen as the final decision-making scheme in this study. In Scheme #24, the location of the new waste transfer station is located at facility alternative points 28 and 30, and 7 roads have been upgraded; the maximizing the minimum NIMBY distance (the first objective function value) of Scheme #26 is 1122 m; the cost of garbage transportation (the second objective function value) is 2959; the cost of construction of waste transfer stations and road upgrades is 2830968. The road upgrading and construction and the location of the new waste transfer station in Model 3 are shown in Fig 8. As Fig 8 demonstrates, the moving direction of garbage to waste transfer station is shown on roads. Table 6 shows the NIMBY distance, garbage transportation cost and construction cost of each population center under the optimization results of Model 3.

**Table 6:** The NIMBY distance and waste transportation cost of population centers in scheme #24 under Model 3.

Population center No.	NIMBY distance(m)	transportation cost	Population center No.	NIMBY distance(m)	transportation cost
1	6412	196	14	1392	12
2	4002	113	15	1953	48
3	1443	48	16	2922	89
4	1372	105	17	4855	86
5	1520	26	18	3663	166
6	2720	233	19	2218	79
7	4118	194	20	2038	132
8	3399	13	21	1122	27
9	4393	352	22	2199	33
10	3375	126	23	2407	66
11	6912	72	24	1499	173
12	1891	38	25	7004	197
13	2073	85	26	5430	250

#### 4.3.2 Results analysis

**Fig 9:**
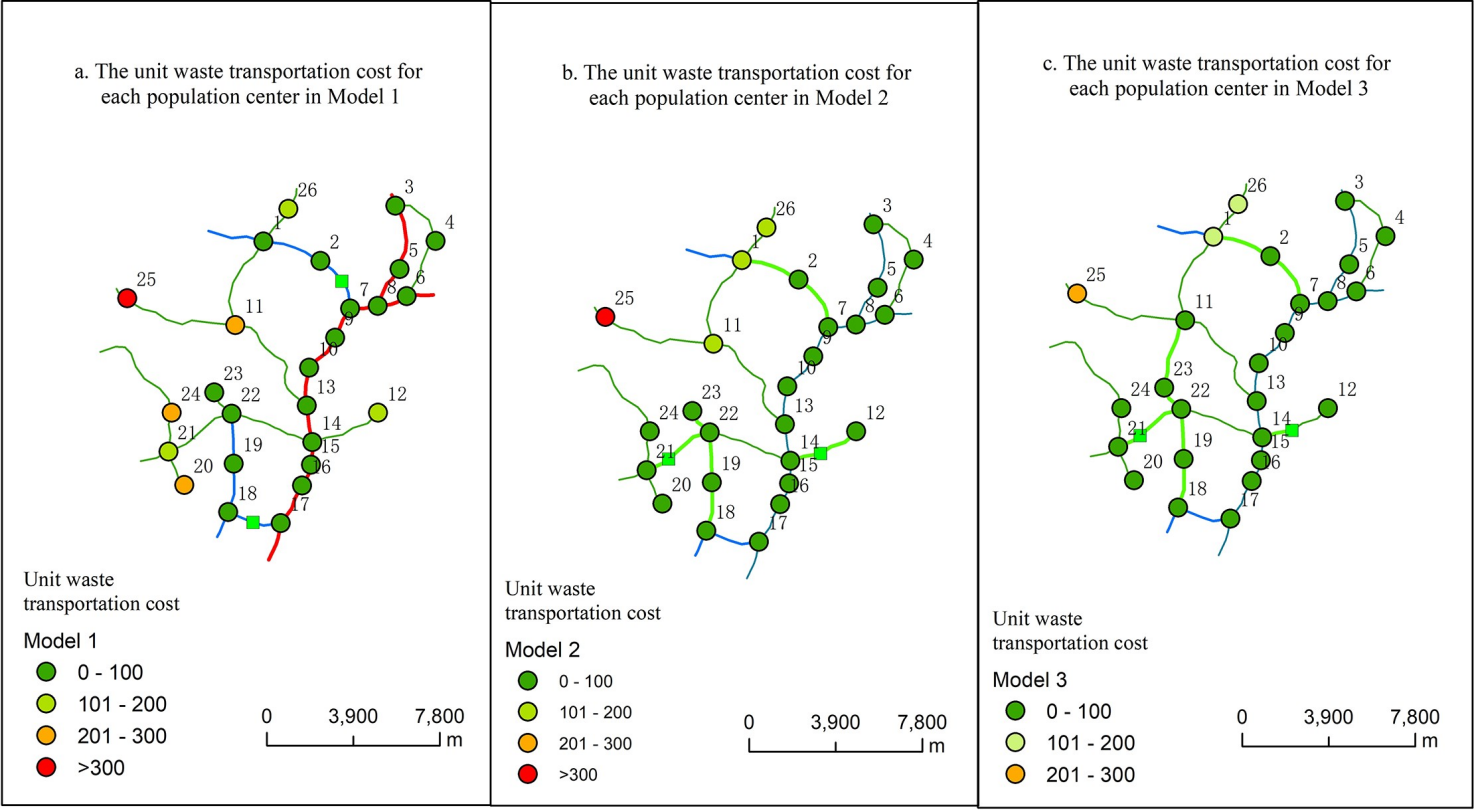
Spatial distribution of waste transportation costs in various settlements under various scenarios.

To further compare and analyze the calculated results of the three models, we divided the unit waste transportation cost calculated by Model 1, Model 2 and Model 3 into four grades (0–100, 101-200h, 201–300 and >300), respectively (as shown in Fig 9). The results of Model 1 calculation are shown in Fig 9(A). Fig 9(A) shows the following information: (1) the unit waste transportation cost in 19 population centers is less than 100, and the waste output of these 19 population centers accounts for 74.6% of the total output of the town; (2) there are 3 population centers with the unit waste transportation cost ranging from 100 to 200, the waste output of these 3 population centers accounts for 10.2% of the total output of the town; (3) the unit waste transportation cost of 4 population centers is more than 200, and the waste output of these 4 population centers accounts for 15.2% of the total output of the town.

The results of Model 2 calculation are shown in Fig 9(B). Fig 9(B) shows the following information: (1) the unit waste transportation cost in 22 population centers is less than 100, and the waste output of these 22 population centers accounts for 90.6% of the total output of the town; (2) there are 3 population centers with the unit waste transportation cost ranging from 100 to 200, the waste output of these 3 population centers accounts for 7.9% of the total output of the town; (3) the unit waste transportation cost of 1 population centers is more than 200, and the waste output of this population centers accounts for 1.5% of the total output of the town.

The results of Model 3 calculation are shown in Fig 9(C). Fig 9(C) shows the following information: (1) the unit waste transportation cost in 3 population centers is more than 200, and the waste output of these 3 population centers accounts for 7.6% of the total output of the town; (2) there are 3 population centers with the unit waste transportation cost ranging from 100 to 200, the waste output of these 3 population centers accounts for 7.9% of the total output of the town; (3) there is no red dot in the figure, which indicates that the unit waste transportation cost of each population center is less than 300.

Overall, compared with the calculation results of Model 1, the number of green spots in Model 2 increased significantly, and the unit waste transportation cost of some population centers have been significantly reduced. Compared with the calculation results of Model 2, the waste transportation cost of Model 3 is further reduced. Therefore, improvement of traffic conditions can effectively reduce the unit waste transportation costs for rural residents.

## 5 Conclusion

Scientifically planning the location of waste transfer stations is an important way to reduce the waste transportation costs, especially for rural areas. Therefore, the purpose of this study is to ensure the minimum Euclidean distance between the waste transfer station and the population center is the maximum, the garbage transportation cost of each population center, construction costs for waste transfer stations, construction and upgrade costs for roads on a traffic network. An optimization mixed-integer linear programming model and the IMOSA algorithm for location optimization of waste transfer stations in rural areas was investigated. A practical case study is presented in detail to illustrate the application of the proposed mathematical model, the accessibility of waste transfer stations in the rural areas of China was explored. The research results show that the IMOSA algorithm can effectively solve the location problem of waste transfer stations in rural areas. In addition, the traffic network has an important influence on the optimization of the locations of waste transfer stations in rural areas. The improvement of traffic conditions can effectively reduce the cost of waste transportation, improve the efficiency of waste transportation and improve the quality of life for rural residents.

## Supporting information

S1 Table The corresponding scheme and objective function value of Pareto optimal solution of Model 2. (DOCX)

S2 Table The corresponding scheme and objective function value of Pareto optimal solution of Model 3. (DOCX)

S1 Data (RAR)
