# Supplementary material for: Optimizing locations of waste transfer stations in rural areas
Source: PLoS One. 2021 May 21;16(5):e0250962. doi: 10.1371/journal.pone.0250962 (PMC8139517; doi:10.1371/journal.pone.0250962)
Supplement: S1 Table — (DOCX) [file pone.0250962.s001.docx]

**A1 Table.** **The corresponding scheme and objective function value of Pareto optimal solution of Model 2.**

| Solution | Number of new facilities | Location of new facilities | Obj1 | Obj2 | Obj3 |
| --- | --- | --- | --- | --- | --- |
| #1 | $p=1$ | 28 | 1122 | 3419 | 3636280 |
| #2 | $p=1$ | 35 | 1048 | 3635 | 3433128 |
| #3 | $p=1$ | 31 | 1211 | 3791 | 3240760 |
| #4 | $p=1$ | 28 | 1122 | 3559 | 3068040 |
| #5 | $p=1$ | 35 | 1048 | 3943 | 2520088 |
| #6 | $p=1$ | 26 | 1122 | 3774 | 2451620 |
| #7 | $p=1$ | 31 | 1211 | 4100 | 2327720 |
| #8 | $p=1$ | 35 | 1048 | 4187 | 1903668 |
| #9 | $p=1$ | 28 | 1122 | 4225 | 1626468 |
| #10 | $p=1$ | 31 | 1211 | 4460 | 1699860 |
| #11 | $p=1$ | 28 | 1122 | 4628 | 1182212 |
| #12 | $p=1$ | 35 | 1048 | 4946 | 1355852 |
| #13 | $p=1$ | 31 | 1211 | 5073 | 1163484 |
| #14 | $p=1$ | 35 | 1048 | 5754 | 899436 |
| #15 | $p=1$ | 31 | 1211 | 5514 | 826268 |
|  |  |  |  |  |  |
| #16 | $p=2$ | 28,34 | 739 | 2334 | 3805448 |
| #17 | $p=2$ | 28,30 | 1122 | 2762 | 3721528 |
| #18 | $p=2$ | 31,33 | 1211 | 3130 | 3712984 |
| #19 | $p=2$ | 29,31 | 1211 | 3451 | 3642360 |
| #20 | $p=2$ | 28,34 | 739 | 2534 | 3018648 |
| #21 | $p=2$ | 28,30 | 1122 | 3070 | 2808488 |
| #22 | $p=2$ | 29,31 | 1211 | 3652 | 2855560 |
| #23 | $p=2$ | 31,33 | 1211 | 3826 | 2804924 |
| #24 | $p=2$ | 28,34 | 739. | 2894 | 2350788 |
| #25 | $p=2$ | 28,34 | 739 | 3279 | 2223012 |
| #26 | $p=2$ | 28,30 | 1122 | 3201 | 2312728 |
| #27 | $p=2$ | 29,31 | 1211 | 3913 | 2312600 |
| #28 | $p=2$ | 31,33 | 1211 | 4136 | 2276924 |
| #29 | $p=2$ | 28,34 | 739 | 3504 | 1849252 |
| #30 | $p=2$ | 28,30 | 1122 | 3522 | 2077912 |
| #31 | $p=2$ | 31,33 | 1211 | 4533 | 1772024 |
| #32 | $p=2$ | 29,31 | 1211 | 4143 | 2052724 |
| #33 | $p=2$ | 28,30 | 1122 | 3714 | 1714312 |
| #34 | $p=2$ | 31,33 | 1211 | 4727 | 1427224 |
| #35 | $p=2$ | 29,31 | 1211 | 5374 | 1319228 |
|  |  |  |  |  |  |
| #36 | $p=3$ | 30,31,32 | 1211 | 3071 | 4373744 |
| #37 | $p=3$ | 28,33,34 | 739 | 2015 | 4193752 |
| #38 | $p=3$ | 29,31,32 | 1211 | 3249 | 3971744 |
| #39 | $p=3$ | 28,29,31 | 1122 | 2799 | 4062176 |
| #40 | $p=3$ | 30,31,32 | 1211 | 3271 | 3970944 |
| #41 | $p=3$ | 28,33,34 | 739 | 2215 | 3406952 |
| #42 | $p=3$ | 29,31,32 | 1211 | 3449 | 3568944 |
| #43 | $p=3$ | 30,31,32 | 1211 | 3532 | 3562784 |
| #44 | $p=3$ | 28,29,31 | 1122 | 2999 | 3275376 |
| #45 | $p=3$ | 28,33,34 | 739 | 2328 | 3034152 |
| #46 | $p=3$ | 28,33,34 | 739 | 2536 | 2659256 |
| #47 | $p=3$ | 29,31,32 | 1211 | 3962 | 3029242 |
| #48 | $p=3$ | 28,29,31 | 1122 | 3226 | 2742716 |
| #49 | $p=3$ | 30,31,32 | 1211 | 3904 | 3083184 |
| #50 | $p=3$ | 29,31,32 | 1211 | 3710 | 3160784 |
| #51 | $p=3$ | 28,29,31 | 1122 | 3492 | 2472540 |
| #52 | $p=3$ | 28,33,34 | 739 | 2808 | 2222936 |
| #53 | $p=3$ | 29,31,32 | 1211 | 4150 | 2814202 |
| #54 | $p=3$ | 28,29,31 | 1122 | 3729 | 2156060 |
| #55 | $p=3$ | 30,31,32 | 1211 | 4257 | 2765246 |
