# Supplementary material for: Optimizing locations of waste transfer stations in rural areas
Source: PLoS One. 2021 May 21;16(5):e0250962. doi: 10.1371/journal.pone.0250962 (PMC8139517; doi:10.1371/journal.pone.0250962)
Supplement: S2 Table — (DOCX) [file pone.0250962.s002.docx]

**A2 Table.** **The corresponding scheme and objective function value of Pareto optimal solution of Model 3**

| Solution | Number of new facilities | Location of new facilities | Obj1 | Obj2 | Obj3 |
| --- | --- | --- | --- | --- | --- |
| #1 | $p=1$ | 28 | 1122 | 3112 | 4993384 |
| #2 | $p=1$ | 31 | 1211 | 3316 | 3707264 |
| #3 | $p=1$ | 28 | 1122 | 3288 | 3365304 |
| #4 | $p=1$ | 30 | 1372 | 3446 | 3529240 |
| #5 | $p=1$ | 31 | 1211 | 3624 | 2794224 |
| #6 | $p=1$ | 30 | 1372 | 3646 | 2742440 |
| #7 | $p=1$ | 28 | 1122 | 3654 | 2364884 |
| #8 | $p=1$ | 31 | 1211 | 3984 | 2166364 |
| #9 | $p=1$ | 30 | 1372 | 3952 | 2181620 |
| #10 | $p=1$ | 30 | 1372 | 4337 | 2053593 |
| #11 | $p=1$ | 31 | 1211 | 4090 | 1799164 |
| #12 | $p=1$ | 31 | 1211 | 4278 | 1584124 |
| #13 | $p=1$ | 30 | 1372 | 4931 | 1674977 |
| #14 | $p=1$ | 28 | 1122 | 6343 | 1380292 |
| #15 | $p=1$ | 28 | 1122 | 6521 | 1170852 |
|  |  |  |  |  |  |
| #16 | $p=2$ | 28,30 | 1122 | 2611 | 3894968 |
| #17 | $p=2$ | 31,33 | 1211 | 3018 | 3822048 |
| #18 | $p=2$ | 29,31 | 1211 | 3069 | 3755744 |
| #19 | $p=2$ | 29,30 | 1372 | 3872 | 3755744 |
| #20 | $p=2$ | 28,30 | 1122 | 2752 | 3326728 |
| #21 | $p=2$ | 31,33 | 1211 | 3218 | 3035248 |
| #22 | $p=2$ | 29,31 | 1211 | 3270 | 2968944 |
| #23 | $p=2$ | 29,30 | 1372 | 4072 | 2968944 |
| #24 | $p=2$ | 28,30 | 1122 | 2959 | 2830968 |
| #25 | $p=2$ | 31,33 | 1211 | 3469 | 2656608 |
| #26 | $p=2$ | 29,30 | 1372 | 4348 | 2593004 |
| #27 | $p=2$ | 28,30 | 1122 | 3204 | 2596152 |
| #28 | $p=2$ | 29,31 | 1211 | 3578 | 2459344 |
| #29 | $p=2$ | 29,30 | 1372 | 4875 | 2370940 |
| #30 | $p=2$ | 29,30 | 1372 | 4741 | 2243164 |
| #31 | $p=2$ | 28,30 | 1122 | 3396 | 2232552 |
| #32 | $p=2$ | 31,33 | 1211 | 3673 | 2282848 |
| #33 | $p=2$ | 29,31 | 1211 | 3809 | 2199468 |
| #34 | $p=2$ | 31,33 | 1211 | 3908 | 1810272 |
| #35 | $p=2$ | 29,31 | 1211 | 4273 | 1703228 |
|  |  |  |  |  |  |
| #36 | $p=3$ | 30,31,32 | 1211 | 2096 | 4757744 |
| #37 | $p=3$ | 28,30,32 | 1122 | 1914 | 4598488 |
| #38 | $p=3$ | 29,31,32 | 1211 | 2274 | 4355744 |
| #39 | $p=3$ | 28,33,34 | 739 | 2015 | 4193752 |
| #40 | $p=3$ | 30,31,32 | 1211 | 2296 | 3970944 |
| #41 | $p=3$ | 28,30,32 | 1122 | 2114 | 3811688 |
| #42 | $p=3$ | 28,33,34 | 739 | 2215 | 3406952 |
| #43 | $p=3$ | 28,30,32, | 1122 | 2293 | 3567848 |
| #44 | $p=3$ | 29,31,32 | 1211 | 2474 | 3568944 |
| #45 | $p=3$ | 30,31,32 | 1211 | 2557 | 3562784 |
| #46 | $p=3$ | 28,30,32 | 1122 | 2483 | 3327752 |
| #47 | $p=3$ | 28,33,34 | 739 | 2328 | 3034152 |
| #48 | $p=3$ | 29,31,32 | 1211 | 2735 | 3160784 |
| #49 | $p=3$ | 30,31,32 | 1211 | 2929 | 3083184 |
| #50 | $p=3$ | 29,31,32 | 1211 | 2987 | 3029242 |
| #51 | $p=3$ | 28,30,32 | 1122 | 2713 | 3038792 |
| #52 | $p=3$ | 29,31,32 | 1211 | 3175 | 2814202 |
| #53 | $p=3$ | 30,31,32 | 1211 | 3282 | 2765246 |
| #54 | $p=3$ | 28,33,34 | 739 | 2536 | 2659256 |
| #55 | $p=3$ | 28,33,34 | 739 | 2808 | 2222936 |
